# Decrypting the molecular basis of cellular drug phenotypes by dose-resolved expression proteomics

**DOI:** 10.1038/s41587-024-02218-y

**Published:** 2024-05-07

**Authors:** Stephan Eckert, Nicola Berner, Karl Kramer, Annika Schneider, Julian Müller, Severin Lechner, Sarah Brajkovic, Amirhossein Sakhteman, Christian Graetz, Jonas Fackler, Michael Dudek, Michael W. Pfaffl, Percy Knolle, Stephanie Wilhelm, Bernhard Kuster

**Affiliations:** 1 Chair of Proteomics and Bioanalytics, School of Life Sciences, Technical University of Munich, Freising, Germany; 2 German Cancer Consortium (DKTK), partner site Munich, a partnership between DKFZ and University Center Technical University of Munich, Munich, Germany; 3 German Cancer Research Center (DKFZ), Heidelberg, Germany; 4 Chair of Animal Physiology and Immunology, School of Life Sciences, Technical University of Munich, Freising, Germany; 5 Institute of Molecular Immunology and Experimental Oncology, School of Medicine and Health, Technical University of Munich, Munich, Germany

**Keywords:** Proteomics, Chemical biology

## Abstract

Proteomics is making important contributions to drug discovery, from target deconvolution to mechanism of action (MoA) elucidation and the identification of biomarkers of drug response. Here we introduce decryptE, a proteome-wide approach that measures the full dose–response characteristics of drug-induced protein expression changes that informs cellular drug MoA. Assaying 144 clinical drugs and research compounds against 8,000 proteins resulted in more than 1 million dose–response curves that can be interactively explored online in ProteomicsDB and a custom-built Shiny App. Analysis of the collective data provided molecular explanations for known phenotypic drug effects and uncovered new aspects of the MoA of human medicines. We found that histone deacetylase inhibitors potently and strongly down-regulated the T cell receptor complex resulting in impaired human T cell activation in vitro and ex vivo. This offers a rational explanation for the efficacy of histone deacetylase inhibitors in certain lymphomas and autoimmune diseases and explains their poor performance in treating solid tumors.

## Abstract (web summary)

Proteome-wide measurements of dose–response curves for 144 drugs reveal drug mechanisms of action.

## Main

Most drugs act on proteins^1,2^ and it has been known since the days of Paracelsus that drugs exert their effects in a dose-dependent fashion. The molecular processes leading to a drug-induced change in cellular phenotype can be roughly divided into (1) target binding, (2) pathway engagement and (3) cellular reprogramming to arrive at a new viable state or cell death, together forming the MoA of a drug^2,3^. Today, quantitative mass spectrometry is the most comprehensive approach for the proteome-wide characterization of drugs on all three levels because of its ability to assay thousands of proteins in complex cellular backgrounds in parallel^2^. The technology does not require any preconceived hypotheses as to which proteins a drug may target, which pathways it may perturb, or what the proteomic composition of the new cellular state may be. While phenotypic dose–response measurements have been commonplace for decades in pharmacology, there is a lack of proteomic studies that consider dose as the arguably most important characteristic of a drug.

Potent drugs typically engage their cellular targets within minutes, sometimes hours if they have a particularly slow on-rate^4,5^. Among the most successful approaches for proteome-wide target deconvolution are activity- and affinity-based proteome profiling. Both aim to measure the interaction of a drug with its target(s) directly^6–9^. When performed in a dose-dependent fashion, they also allow the determination of apparent interaction constants^10,11^. Alternative methods measure drug-induced changes in other biophysical or biochemical properties of proteins such as solubility at elevated temperature^12,13^ or in the presence of organic solvents^14,15^, sensitivity to oxidizing reagents^16^ or susceptibility to partial enzymatic hydrolysis^17,18^. While powerful, these methods often require high levels of target engagement to lead to measurable effects. In addition, the observed effects often extend beyond the target itself, thus complicating the differentiation between direct and indirect drug effects.

Because many cellular pathways are regulated by reversible posttranslational protein modifications (PTMs), mass spectrometry can also be used to measure if a drug engages pathways downstream of the target^19^. The time frame here is also typically in the minute to few hours range^19^. Published studies often report large numbers of observable PTM changes as a result of applying arbitrary and typically high single doses of a drug or because the data were collected after many hours of treatment^20^. Again, the interpretation of such data can be difficult because both direct and indirect effects are contained in the data. It has only very recently been demonstrated that measuring drug effects on PTMs in a dose- and time-dependent fashion is a more powerful approach to pathway engagement measurements because it enables prioritizing the data by drug potency^19^.

The adaptation of a cell to a new functional state in response to a drug is a complex process, often involving changes in gene expression, messenger RNA (mRNA) and/or protein stabilization or degradation over the course of several hours or even days^2^. The L-1000 connectivity map project^21^ has addressed the transcriptional angle of drug perturbations and, more recently, a number of studies have extended such investigations to the level of the proteome^22–24^. Such data are useful because they characterize the molecular consequences that underlie the cellular endpoint (phenotype) of a drug treatment. However, to the best of our knowledge, a systematic evaluation of the dose–response characteristics of drug-induced proteome expression changes has not been undertaken yet, limiting insights as to the molecular basis that drive and describe the observed phenotypic changes.

Here, we close this gap by introducing a method termed decryptE, able to measure the dose–response characteristics of expression changes of ~8,000 proteins in human cells in response to a drug. We exemplify the feasibility and utility of the approach by characterizing 144 drugs with diverse MoA and highlighting several noteworthy findings including the repression of (Jurkat) T cell activation in response to histone deacetylase (HDAC) inhibitors. The collective data comprise >1 million dose–response curves that are accessible via ProteomicsDB and the custom-built decryptE web application for further exploration.

## Results

### DecryptE for dose-resolved expression proteomics

**Fig. 1:**
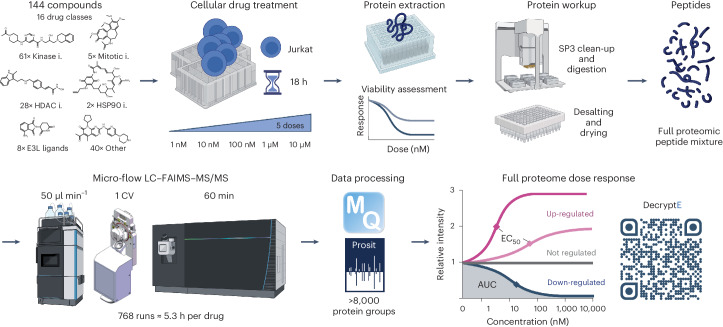
DecryptE workflow for the proteome-wide and dose-dependent characterization of drug-induced protein expression changes. See text and methods for details (i, inhibitor; E3L, E3 ligase; AUC, area under the curve).

**Extended Data Fig. 1:**
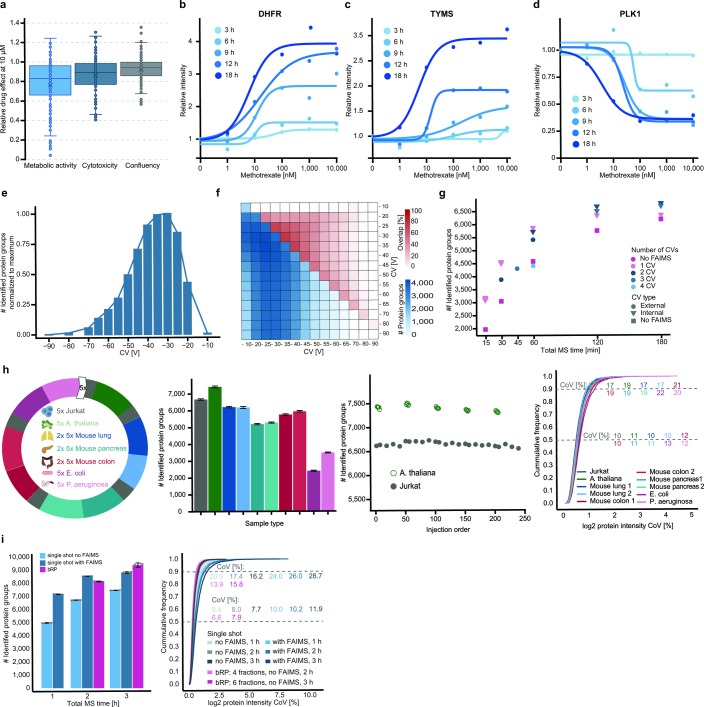
Optimization and characterization of the decryptE workflow for the profiling of drug induced expression changes at scale. **a**) Boxplot showing the distribution of drug effect at 10,000 nM for all three measures of cell fitness. Each dot represents a drug (n = 144). Horizontal lines, boxes and whiskers of the boxplot depict the median, the range between the second and the third quartile and the 1.5-fold interquartile range. **b**) Dose-response curves of DHFR following treatment with Methotrexate for different times. **c**) Same as panel b) but for TYMS. **d**) Same as panel b) but for PLK1. **e**) Distribution of the relative number of identified protein groups from Jurkat cells as a function of the applied FAIMS compensation voltage (CV). **f**) Heatmap comparing the number and overlap of identified protein groups between any combination of two CVs (same data as in panel e). **g**) Number of identified protein groups from Jurkat cells as a function of the total LC-MS/MS time used per sample. **h**) Far left panel: schematic representation of the experimental design for testing the robustness of the micro-flow LC-FAIMS-MS/MS method. Colors represent the different sample types and the size of the ring segment is relative to the number of analyses in each segment (total of 250 samples analysed). Middle left panel: bar plot summarizing the number of proteins identified for each sample type. Error bars represent mean ± standard deviation (SD, n = 25 technical replicates for each sample type). Middle right panel: number of identified protein groups plotted as a function of the consecutive order in which the samples were analysed. Far right panel: Cumulative density plot summarizing the precision with which proteins were quantified across replicate experiments. Dotted lines indicate the respective fraction of proteins (50% and 90%) that were quantified with the given coefficient of variation (CoV). **i**) Left panel: Bar plot showing the number of identified proteins by single shot micro-flow LC MS/MS with or without FAIMS installed or by micro-flow LC-MS/MS after fractionation using high pH reversed phase chromatography (4 or 6 fractions) and using the specified amount of analysis time. Data are average values ± SD from n = 4 technical replicates. Right panel: same as panel h (far right, but for the data shown in the left panel of i).

**Extended Data Fig. 2:**
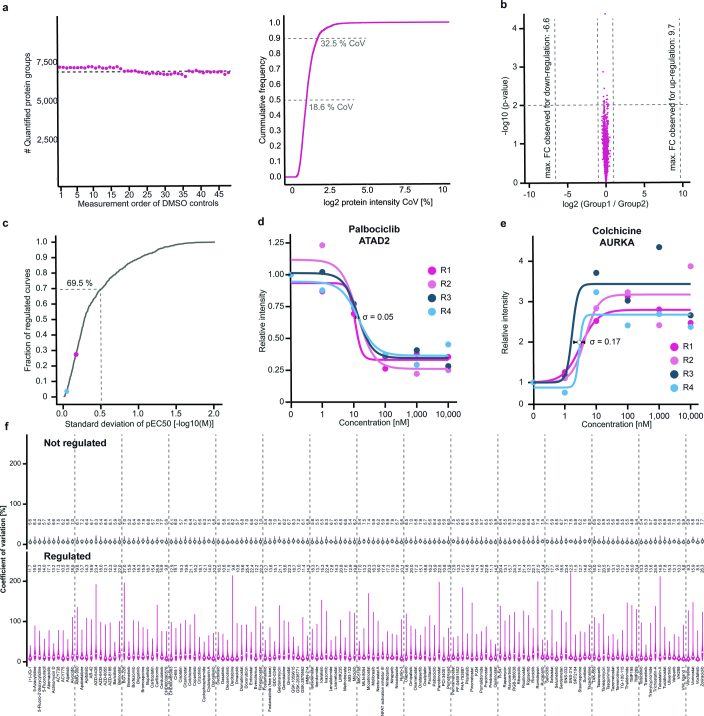
Reproducibility assessment of the decryptE workflow. **a**) Left panel: number of quantified protein groups from DMSO control samples that were analyzed along the entire time frame of the proteomic screen plotted as a function of the consecutive order in which the samples were analyzed. Right panel: Cumulative density plot summarizing the precision with which proteins were quantified across DMSO control samples. Dotted lines indicate the respective fraction of proteins (50% and 90%) that were quantified with the given coefficient of variation (CoV). **b**) Volcano plot analysis for n = 4694 protein groups from 48 DMSO control samples from the proteomic screen which were randomly assigned to two groups. Analysis of significance was done using a two-sided Welch’s t-test without multiple testing correction. **c**) Cumulative density plot showing the reproducibility of pEC50 estimations from replicate dose-response analysis (n = 4) of palbociclib, panobinostat, and colchicine. 69.5 % of all pEC50 estimates were reproducible within ½ log10 step of drug concentration. Blue and pink dots indicate the SD for example curves of panel d) and e) respectively. **d**) Replicate dose-response curves of ATAD2 regulated by palbocicblib along with the SD for the pEC50. **e**) Same as panel d) but for AURKA regulated by colchicine. **f**) Upper panel: Violine plots showing the distribution of CoV of all proteins which were not regulated by drug treatment for each of the 144 drugs. Median values are given above each violine for each drug. Lower panel: same as upper panel but for all proteins that showed drug induced expression changes.

The decryptE approach (Fig. 1) was developed using Jurkat acute T cell leukemia cells as a model system and is exemplified by analyzing 144 drugs from 16 drug classes (Supplementary Table 1). These comprise approved (53) and phase III (15) drugs as well as phase I/II investigational or frequently used tool compounds (76). Briefly, cells were grown in 48-well plates and treated for 18 hours with five drug doses in full log_10_ steps between 1 and 10,000 nM and vehicle control (dimethylsulfoxide, DMSO). Metabolic activity and cytotoxicity, as well as cell morphology, were determined for all drugs across the same dose range in parallel and were only marginally affected within the time frame of the experiment (Supplementary Table 1 and Extended Data Fig. 1a) while observed proteomic drug effects were most pronounced (Extended Data Fig. 1b–d). Proteins were extracted by SDS-containing buffer and digested into peptides on a robotic platform following the single-pot, solid-phase-enhanced sample preparation protocol (SP3) approach^25^. We previously demonstrated that microflow-liquid chromatography with tandem mass spectrometry (LC–MS/MS) enables high-throughput proteome measurements^26^ and, here, extended the approach by incorporating an ion mobility dimension (high-field asymmetric ion mobility spectrometry, FAIMS) to achieve a proteome coverage of >7,000 proteins per hour (Extended Data Fig. 1e–i). The entire drug screen required 768 hours of instrument time (equivalent to 5.3 h per drug) and led to the identification and quantification of 8,892 proteins using MaxQuant and Prosit rescoring^27,28^. Based on 48 DMSO replicates, a median quantitative precision of 19% coefficient of variation (CoV) was determined for the assay (Extended Data Fig. 2a) with a high degree of data uniformity (Extended Data Fig. 2b). Dose–response curves were fitted to the data providing information on drug potency (effective concentration required to achieve 50% of the effect, EC_50_) and effect size (area under the curve or fold change over DMSO). The statistical power of the dose–response data enabled robust classification of 1,133,847 dose–response curves (regulated or not) that formed the basis for all further analysis. DecryptE data were reproducible with 69.5% of all determined EC_50_ values within half a log_10_ of drug concentration (Extended Data Fig. 2c–e). Moreover, the CoVs of regulated proteins were invariably higher than for not regulated proteins (Extended Data Fig. 2f).

To facilitate the use of this resource by the community, the data can be explored in ProteomicsDB (https://proteomicsdb.org/decryptE)^29^ as well as in a custom-built Shiny App (https://decrypte.proteomics.ls.tum.de/) in which dose–response curves can be visualized and compared. Additional information on cell morphology, cell metabolic activity, cytotoxicity, protein half-lives and protein targets of compounds and drug-target affinity (where available) are provided to help interpreting the observed effects.

### High-level analysis of decryptE profiles

**Fig. 2:**
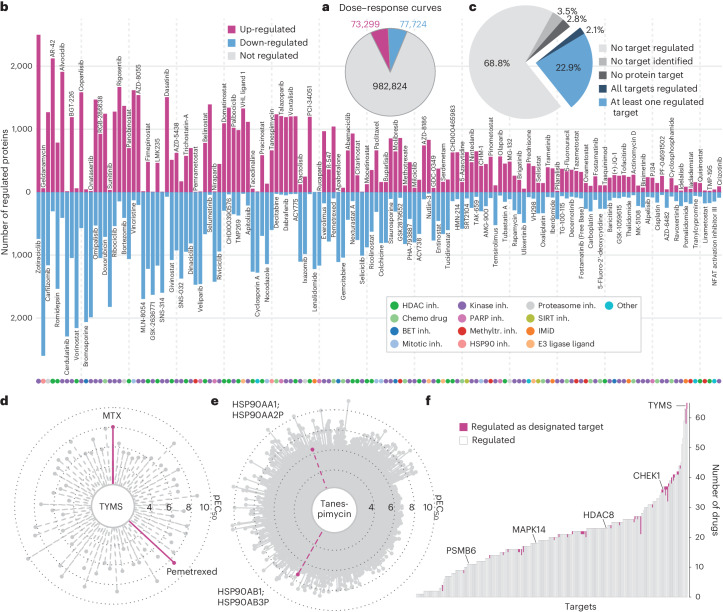
Summary of drug-induced expression changes. **a**, Pie chart of the absolute number and relative distribution of dose–response curve categories. **b**, Bar plot showing the number of up- or down-regulated proteins for each of the 144 drugs (inh., inhibitor; Methyltr., methyltransferase). **c**, Pie chart of the proportion of drugs that did or did not lead to expression changes of at least one designated target protein. **d**, Radar plot showing the number of drugs that changed the expression of the protein TYMS. The length of each line indicates the pEC_50_ (−log_10_ EC_50_) of the observed regulation. MTX and pemetrexed are highlighted because TYMS is a designated target of both drugs. **e**, Same as **d** but showing all proteins that are regulated by the drug Tanespimycin. The highlighted proteins are targets of this drug. **f**, Bar plot showing the number of drugs (*y* axis) that regulate a particular target protein. The proportions of drugs for which a particular protein is a designated target are highlighted in pink.

**Extended Data Fig. 3:**
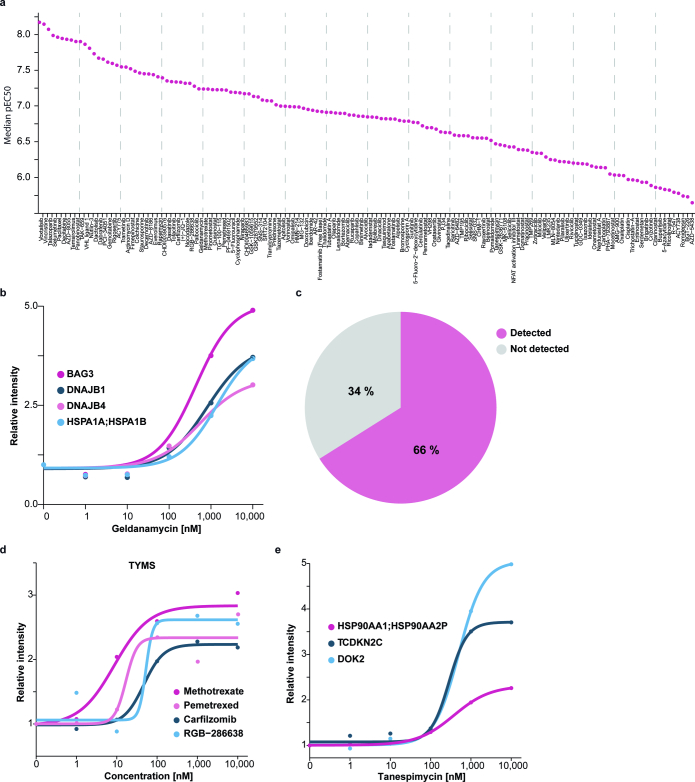
Global and specific analyses of quantitative drug-induced protein expression changes. **a**) List of all drugs ranked by the median potency (expressed as pEC50 = −log10 EC50) with which they regulated protein expression in Jurkat cells. **b**) Examples of dose-response curves of proteins from cells treated with the HSP90 inhibitor geldanamycin. **c**) Pie chart showing the proportion of all drug targets which are detected in the dataset. **d**) Examples of dose-response curves for drugs that regulated the expression of TYMS. **e**) Same as panel b) but for the HSP90 inhibitor tanespimycin.

Several observations were immediately apparent from a global analysis of the data. First, the abundance of most proteins did not change in response to any drug within the time frame of the experiment (18 h; *n* = 982,824 dose–response curves; 87%) (Fig. 2a). Dose-dependent up-regulation occurred in 73,299 cases and dose-dependent down-regulation was observed in 77,724 cases. Second, the extent to which any of the 144 drugs remodeled the proteome of Jurkat cells varied tremendously. Some drugs regulated the expression of >1,000 proteins, others just a few (Fig. 2b). Similarly, some drugs showed very potent effects, others only at high concentrations (Extended Data Fig. 3a). Both aspects are important to be able to attribute the observed phenotypic (here morphology, metabolic activity and cytotoxicity) and molecular (here protein expression) response of a cell to the MoA of a particular compound. As one might expect, drugs targeting basic cellular processes caused many changes. For instance, HDAC inhibitors such as vorinostat or panobinostat alter transcriptional programs and, consequently, the expression of many proteins. The proteasome inhibitor carfilzomib also showed massive effects because it inhibits a major protein degradation machine in cells. Many changes were also observed for the HSP90 inhibitor geldanamycin because it inactivates a key member of the protein folding machinery. More specifically, geldanamycin strongly up-regulated proteins (up to 50-fold) involved in the unfolded protein response (for example, DNAJB1, HSPA1B) presumably because of a cellular attempt to counter the drug-induced loss of protein folding capacity (Extended Data Fig. 3b). In stark contrast, some drugs induced only minor proteomic changes. Among these were the histone lysine methyltransferase inhibitor lirametostat or the dual c-MET and ALK kinase inhibitor crizotinib. The former suggests that interfering with dynamic histone lysine methylation in cultured Jurkat cells did not bear any consequences within the time frame of the experiment and the latter implies that the viability of Jurkat cells is not dependent on ALK and MET activity.

It is often stated that drug-induced proteome expression changes can be used for the deconvolution of drug targets^12,22,24^. An important learning point from the global decryptE data analysis is that this is generally not the case. First, while the list of 8,892 detected proteins contains 66% of all designated targets of the drugs investigated here (expression regulated or not, Extended Data Fig. 3c), known targets for about 34% of all drugs were missing and simulation showed that this number rapidly increased as proteome coverage decreased (Supplementary Fig. 1). Second, only about 25% of all drugs changed the expression levels of their designated protein target(s) (Fig. 2c). Third, even if that occurred, the particular drug target did often not stand out from the data in terms of potency or effect size, as illustrated by thymidylate synthetase (TYMS). While the protein was up-regulated by its direct binders methotrexate (MTX) and pemetrexed (Extended Data Fig. 3d), TYMS levels were also regulated by 63 other compounds that are not reported to target TYMS (Fig. 2d). Another example is the HSP90 inhibitor tanespimycin. HSP90 levels were regulated by the drug (Extended Data Fig. 3e), but so were hundreds of other proteins and many more potently and with larger effect sizes than HSP90 itself (Fig. 2e). When generalizing this analysis, far more often than not, a protein showed drug-induced expression changes even though it is not the target of that drug (Fig. 2f). Therefore, it seems unlikely that a drug target can generally or clearly be delineated from drug-induced protein expression changes alone.

### Multi-omics analysis of drug-induced cellular remodeling

**Fig. 3:**
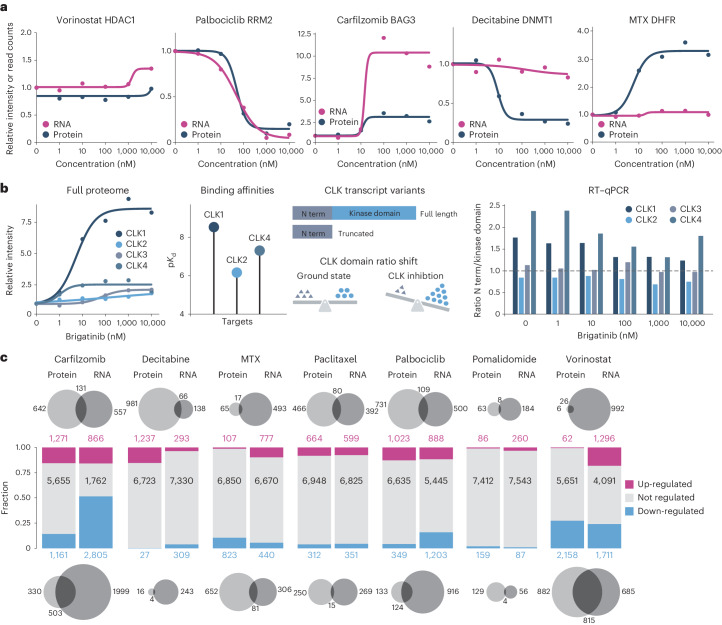
Molecular mechanisms underlying drug-induced protein expression changes measured by decryptE profiling. **a**, Example dose–response curves of drug-induced abundance changes of proteins (blue) and mRNA (pink). **b**, From left to right, the dose–response curves for CLK1–4 following Brigatinib treatment. Binding affinities of Brigatinib and CLK1,2,4 (p*K*_d_ = −log_10_ *K*_d_) determined by kinobead assays^10^. Schematic representation of the two major transcripts for CLK proteins and how the ratio between the two domains shifts to a 1:1 ratio on CLK inhibition. The triangle represents the N-terminal (N term) domain; the dot represents the kinase domain of the protein. Bar plot showing the ratio of the N term and kinase domain transcripts (determined by RT–qPCR) for CLK1–4 as a function of the dose of Brigatinib. **c**, Comparison of drug-induced mRNA and protein expression changes for seven drugs. The bar plots in the middle panel show the fraction of up-, down- or not regulated proteins (left bars) and mRNAs (right bars). The Venn diagrams in the upper panel show the number and overlap of up-regulated proteins versus mRNAs (data confined to mRNAs for which also a protein was detected). The bottom panel shows the same but for down-regulation.

**Extended Data Fig. 4:**
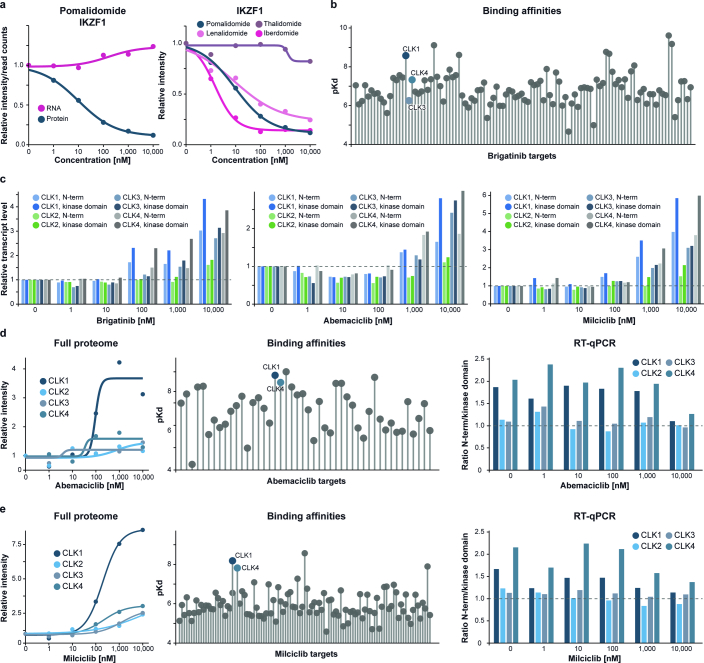
Drug-induced mRNA and protein expression changes. **a**) Dose-response curves of mRNA and protein levels of IKZF1. **b**) Apparent binding affinity constants (pKd = −log10 Kd) of brigatinib-Protein interactions determined by Kinobead competition assays^10^. CLK1,3,4 are marked by respective text. **c**) Left panel: Dose-dependent change of mRNA levels (determined by RT-qPCR) for different CLK1-4 domains following treatment with brigatinib. Middle panel: same as left panel but for abemaciclib. Right panel: same as left panel but for milciclib. **d**) Left panel: dose-response curves of CLK1-4 following treatment with abemaciclib. Middle panel: same as b) but for abemaciclib. Right panel: ratios of the N-terminal and kinase domain transcripts (determined by RT-qPCR) of CLK1-4 in response to abemaciclib. The dotted line marks a 1:1 quantitative ratio of the two mRNAs. **e**) Same as panel d) but for milciclib.

To learn whether drug-induced protein expression changes are rooted in altered transcriptional programs or pre-, co- and/or posttranslational mechanisms, we performed dose-dependent RNA sequencing (RNA-seq) experiments for seven selected drugs in the same cell line and under the same drug treatment conditions. As evident from Fig. 3a, several concordant and discordant effects were observed. For instance, protein and mRNA levels of HDAC1 remained unchanged in response to the HDAC inhibitor vorinostat. In contrast, protein and mRNA levels of the cell cycle regulated protein RRM2 were equipotently diminished in response to the CDK4/6 inhibitor palbociclib. This may be explained by the dose-dependent increase in the number of cells arresting at a stage of the cell cycle where RRM2 levels are low. Conversely, the proteasome inhibitor carfilzomib up-regulated both transcript and protein levels of the cochaperone BAG3 with similar potency but with very different effect sizes, suggesting that BAG3 protein levels only moderately increase in cells on drug treatment. Another scenario is presented by the DNA methyltransferase DNMT1 for which protein but not transcript levels were reduced in response to decitabine. This is in line with literature reporting that decitabine, when integrated into DNA, covalently traps DNA methyltransferases, in turn, leading to their degradation^30^. A similar behavior was observed for molecular glues such as pomalidomide that led to a potent and dose-dependent reduction of the protein IKZF1 but not its mRNA level (Extended Data Fig. 4a). The aforementioned drug MTX led to a strong and dose-dependent increase in protein levels of its direct target DHFR while mRNA levels remained unchanged (Fig. 3a). This clearly points to a posttranscriptional event. Previous in vitro experiments have shown that DHFR binds its own mRNA to repress its translation and that addition of MTX abolishes this repression^31^. This mechanism would be an elegant explanation for the observation that MTX also induces a very strong thermal or solvent stability shifts for DHFR when bound to MTX^12,13,15^.

**Extended Data Fig. 5:**
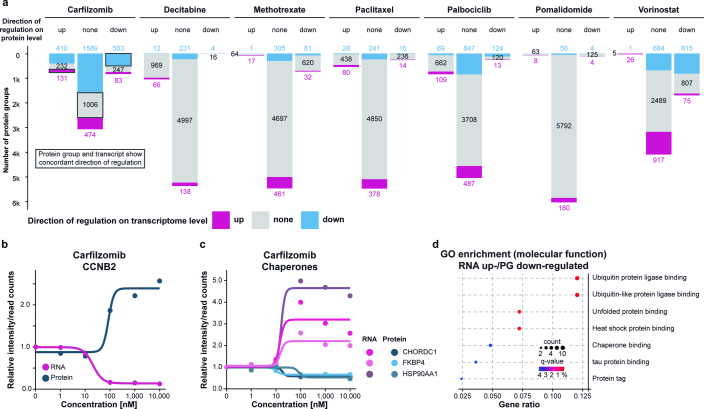
Discrepancies between different omics levels. **a**) Comparison of direction of drug-induced expression changes between mRNA and protein levels for seven drugs. **b**) Dose-response curves of drug-induced abundance changes of CCNB2 on protein (blue) and mRNA (pink) level after carfilzomib treatment. **c**) Same as panel b) but for several proteins of the folding machinery. **d**) GO enrichment of genes that are up-regulated on mRNA and down-regulated on protein level after carfilzomib treatment. Testing of significance was done using the clusterProfiler R package (v. 4.2.2.) with the FDR approach for multiple hypothesis testing correction.

Another case is presented by the dual specificity protein kinases CLK1–4 that showed potent up-regulation of both mRNA and protein levels on treatment with the kinase inhibitors brigatinib, abemaciclib and milciclib (Fig. 3b and Extended Data Fig. 4b–e). Published target deconvolution data showed that these proteins are direct targets of all three drugs^10^. Apart from the full-length protein, CLK1 also exists in two shorter versions that contain the N terminus but lack the kinase domain either owing to intron 4 retention or exon 4 skipping. The different forms of the protein arise from the ability of CLK1 to regulate its own splicing by phosphorylating certain splicing factors^32^. Quantitative PCR with reverse transcription (RT–qPCR) data collected here for CLK1–4 showed that the ratio of N-terminal to full-length transcripts shifted in favor of the full-length transcript at higher drug concentrations, in turn, leading to higher levels of full-length protein. While an interesting observation, it remains unclear whether this has any functional consequences in cells as these drugs block kinase activity at the same time. These selected cases highlight several discrepancies between drug regulated transcriptome and proteome changes that are rooted in different cellular mechanisms. Many more cases are in the data and often with very large drug-specific differences, both in absolute and relative terms (Fig. 3c). It is also apparent from these data that the direction of regulation is not always concordant on mRNA and protein level (Extended Data Fig. 5a). Opposing regulation events are rare for most of the drugs studied. However, carfilzomib treatment up-regulated members of cellular folding machinery on mRNA level while down-regulating the respective proteins (Extended Data Fig. 5b–d), possibly in an attempt to maintain proteostasis.

### Drug response phenotypes group drugs by function

**Fig. 4:**
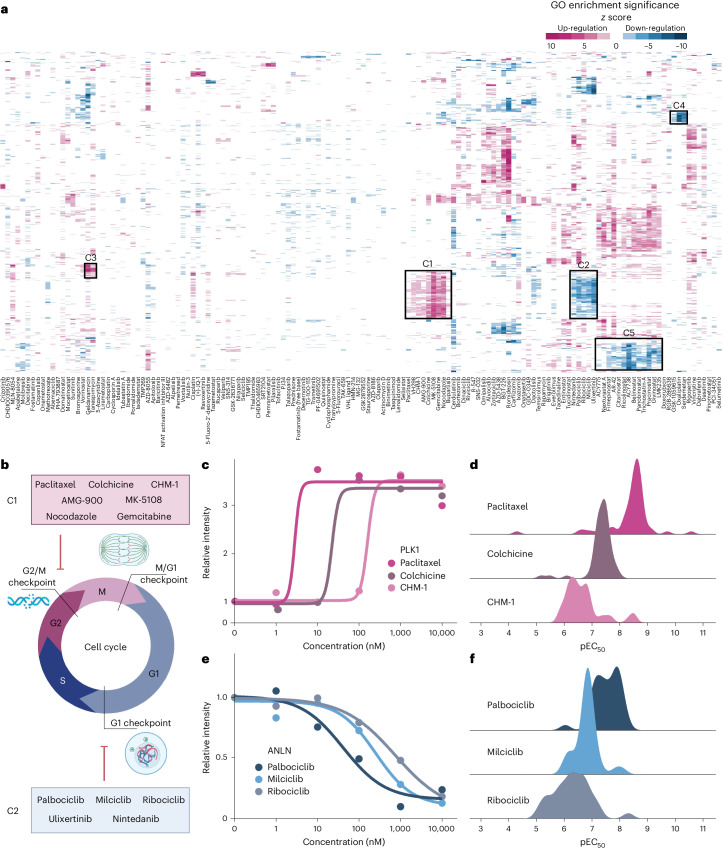
Groups of drugs with similar cellular MoA. **a**, Clustered heatmap of drugs and GO terms enriched by proteins that are up- or down-regulated on drug treatment. **b**, Analysis of drugs in clusters C1 and C2 of **a** showing that the drugs in each cluster similarly affect protein expression at different stages of the cell cycle. **c**, Example dose–response curves for PLK1 and three drugs affecting protein expression at the G2/M checkpoint (cluster C1). **d**, Distribution of the potencies depicted as pEC_50_ (−log_10_ EC_50_) with which each respective drug affects protein expression. **e**, Same as **c** but for ANLN expression and drugs affecting the G1 checkpoint (cluster C2). **f**, Same as **d** but for drugs affecting protein expression at the G1 checkpoint.

While individual drugs may have different targets, they may lead to similar cellular and molecular drug phenotypes^33^. To explore whether decryptE profiles can group drugs in such a way, we performed gene ontology (GO) enrichment analysis for up- or down-regulated proteins for each compound separately, followed by hierarchical clustering of the results for all 144 drugs (Fig. 4a and Supplementary Table 2). Indeed, compounds leading to cell cycle arrest formed two mirrored clusters (C1 and C2) characterized by up- or down-regulation, respectively, of enriched GO terms related to for example sister chromatid separation, mitosis and/or meiosis or cytokinesis. Closer inspection revealed that this analysis distinguished compounds that arrest cells in G1/S or G2/M (Fig. 4b). Examples for proteins that drive this clustering are the strong up- and down-regulation of the cell cycle regulated proteins PLK1 and ANLN, respectively. When summarizing this information for all proteins that are up-regulated or down-regulated, respectively, for all three drugs, they showed a congruent distribution of pEC_50_ values (Fig. 4c–f). On this basis, and when following a guilt-by-association argument, mitotic functions may be assigned to proteins not yet annotated in this process and this concept may hold for other molecular functions present in other clusters. The pEC_50_ plots also ranked drugs by potency identifying paclitaxel as the most potent mitotic inhibitor of the set.

**Extended Data Fig. 6:**
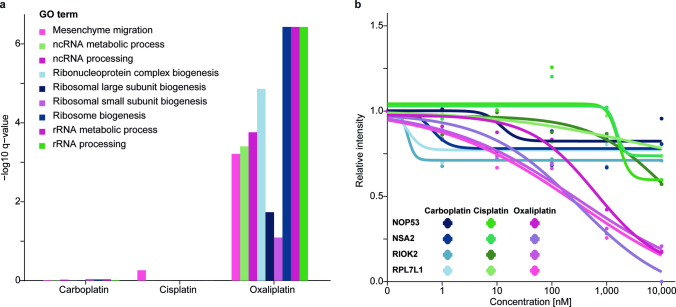
Platinum-based chemo-drugs have different mechanisms of action in cells. **a**) Results of gene ontology (GO) term enrichment analysis for the top nine significantly enriched GO terms of oxaliplatin for all platinum-containing drugs. Testing of significance was done using the clusterProfiler R package (v. 4.2.2.) with the FDR approach for multiple hypothesis testing correction. **b**) Dose-response curves for four example proteins for the same three drugs.

The two aforementioned HSP90 inhibitors formed a small but distinct cluster (C3) driven by GO terms related to the up-regulation of the unfolded protein response (Supplementary Table 2). The PI3K/mTOR inhibitor GSK-1059615, the DNA cross-linker oxaliplatin and the p53 activator serdematan formed a tight cluster indicative of down-regulated ribosome biogenesis (C4) (Supplementary Table 2). The three platinum-containing drugs oxaliplatin, carboplatin and cisplatin did not cluster. And indeed, their decryptE profiles were rather different as exemplified by the down-regulation of ribosomal proteins by oxaliplatin but not the others, implying different cellular modes of action (Extended Data Fig. 6a,b)^34^.

### HDAC inhibitors impair T cell activation

**Fig. 5:**
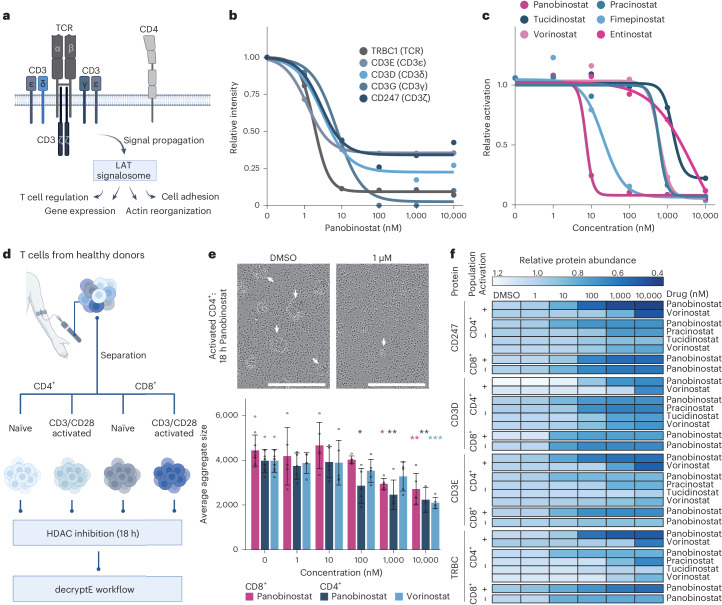
HDAC inhibitors compromise the function of human T cells. **a**, Schematic representation of TCR signaling and cellular outcomes. **b**, Dose-dependent reduction of the expression of TCR components in response to panobinostat in Jurkat cells. **c**, Dose-dependent reduction of activation of Jurkat cells in response to HDAC inhibitors. **d**, Schematic representation of treating human primary T cells with HDAC inhibitors ex vivo. **e**, The upper panels show microscopic pictures of human primary CD4 positive T cells activated by immobilized anti-CD3 and/or CD28 with or without panobinostat treatment (*n* = 1). The lower panel shows a bar plot showing the average size of aggregates (shown in the upper panel) as a function of the applied HDAC inhibitor dose. Error bars indicate the standard deviation from *n* = 5 pictures. *P < 0.05, **P < 0.01, ***P < 0.001 compared to DMSO treatment. Significance testing was done with one-way analysis of variance using *F*-statistics, followed by calculation of Tukey honest significant differences as post hoc test with confidence interval of 95% and correction for multiple comparisons. Scale bars, 400 μm. **f**, Dose-dependent expression changes of proteins in human primary T cells treated ex vivo with HDAC inhibitors.

**Extended Data Fig. 7:**
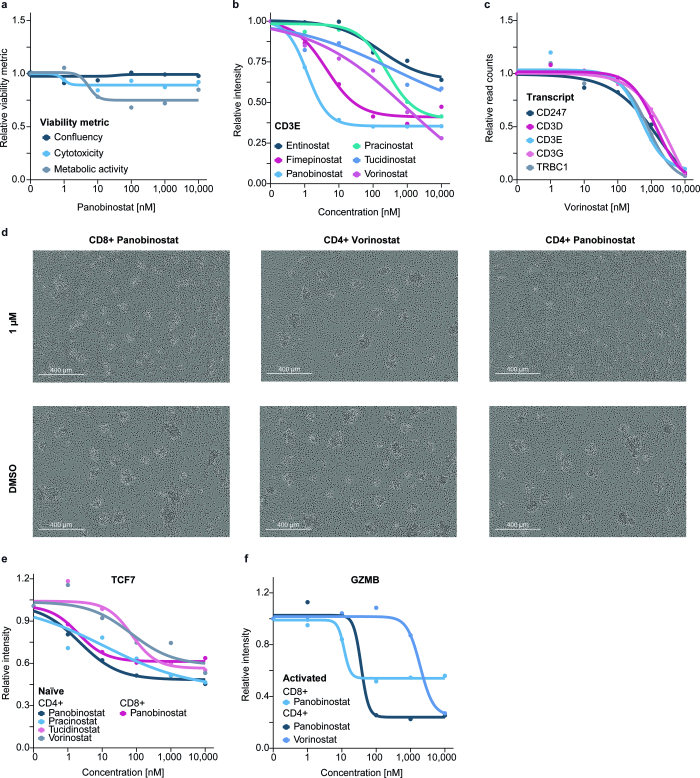
HDAC inhibitors diminish TCR expression in T-cells. **a**) Three measures of cell viability of Jurkat cells treated with panobinostat. **b**) Loss of CD3E expression in response to HDAC inhibitors. **c**) Loss of mRNA expression of several members of the TCR in response to vorinostat. **d**) Microscopic pictures of HDAC inhibitor-treated and CD3/CD28-activated CD8- and CD4- positive primary human T-cells (n = 1). **e**) Dose-dependent reduction of TCF7 protein expression in primary human T-cells (naïve only) in response to HDAC inhibitors. **f**) Dose-dependent reduction of GZMB protein expression in primary human T-cells (activated only).

Unexpectedly, HDAC inhibitors formed a cluster (C5) with strong links to T cell proliferation and activation (Fig. 4a). For instance, panobinostat down-regulated the expression of many key components of the T cell receptor (TCR) with low nanomolar potency (Fig. 5a), notably the TCR itself and its coreceptors (Fig. 5b). Cell viability was only marginally affected within the time frame of the experiment (Extended Data Fig. 7a and Supplementary Table 1). The other HDAC inhibitors showed the same qualitative effect (Extended Data Fig. 7b) and the dose-dependent RNA-seq data collected for vorinostat indicated a concerted transcriptional mechanism rather than protein degradation (Extended Data Fig. 7c). These results demonstrate that the reduction of TCR components can be directly attributed to the loss of HDAC activity. This also resulted in the reduction of anti-CD3 and/or CD28 antibody-mediated T cell activation in genetically engineered Jurkat TCR and/or CD3 effector cells that express luciferase in response to T cell activation (Fig. 5c).

To test whether HDAC inhibition also diminishes protein expression of TCR components in primary human T cells, we separated CD4 and CD8 positive T cells from healthy donors and exposed untreated (referred to as ‘naïve’) and anti-CD3/CD28-activated cells to several HDAC inhibitors (Fig. 5d). Live-cell imaging showed that drug-treated primary cells exhibited a reduced ability to bind to beads carrying anti-CD3/CD28 antibodies (Fig. 5e and Extended Data Fig. 7d). Furthermore, all tested HDAC inhibitors recapitulated the findings of the in vitro Jurkat cell line experiments in all four ex vivo cell populations, exemplified by the dose-dependent loss of CD247, CD3D and CD3E (Fig. 5f). Among many other proteins, a dose-dependent reduction of the transcription factor TCF7, the master regulator of naïve T cell differentiation, was observed in naïve cells after HDAC inhibitor treatment. In activated cells, we observed a reduction of granzyme B levels, an important regulator of T cell activation and proliferation (Extended Data Fig. 7e,f). These results clearly indicate that HDAC inhibition affects T cell activation and differentiation with yet unknown functional consequences, but potentially important ramifications for the use of HDAC inhibitors as anticancer agents or as tools to study T cell biology (Discussion).

## Discussion

DecryptE specifically addresses the longer-term dose-dependent response of a cell to a drug (or other bioactive agent) akin to many phenotypic assays. The difference is that decryptE yields thousands of molecular readouts rather than just one (for example, cell viability or morphology changes). As such, the approach should not be confused with proteomic technologies aiming to elucidate the targets of a drug or illuminating the signaling pathways that lead to a cellular endpoint. These important aspects of drug MoA may be contained in decryptE profiles, but they may not be obvious from the data without substantial previous knowledge. Instead, decryptE profiles reflect the third element of cellular drug MoA, referred to in the introduction, which is the transition of the proteomic makeup to a drug-adapted (new) cellular state. There are two main new technical aspects in the current work. First, showing that the combination of microflow-LC and FAIMS yields deep proteomic coverage and quantitative information (dose–response curves) for one drug and ~8,000 proteins in just over 5 hours of analysis time. Second, demonstrating that dose–response measurements add information not attainable from single doses. The decryptE apporach thus paves the way for large-scale proteome-wide drug perturbation screens that may be further enabled by combining faster and more sensitive mass spectrometers than used here with data independent acquisition or stable isotope multiplexing by tandem mass tags^22,35^. With more than 1 million dose–response curves, our data already provide a rich resource for the scientific community that can be analyzed in many ways not covered here. For instance, we strictly only considered sigmoidal dose–response characteristics because these are regarded as the best understood drug–protein interactions. However, the data may also contain nonsigmoidal drug-induced behaviors that may, for example, represent pharmacological switches in a cell.

While decryptE profiles faithfully report changes in protein expression in response to a drug, these may arise by several mechanisms that add important information regarding drug MoA. In light of the comparisons made here between mRNA and protein–drug profiles, we propose to measure transcriptomes and proteomes systematically in a dose-dependent fashion in parallel in the future to better understand to what extent transcription itself or splicing events play a role. Similarly, adding proteomic measurements that address protein synthesis and degradation, for example by pulse-labeling using stable isotopes^36,37^, will provide additional important insights. The latter is particularly important given the high attention in current drug discovery to the development of chemical degrader molecules such as proteolysis targeting chimeras or molecular glues.

**Extended Data Fig. 8:**
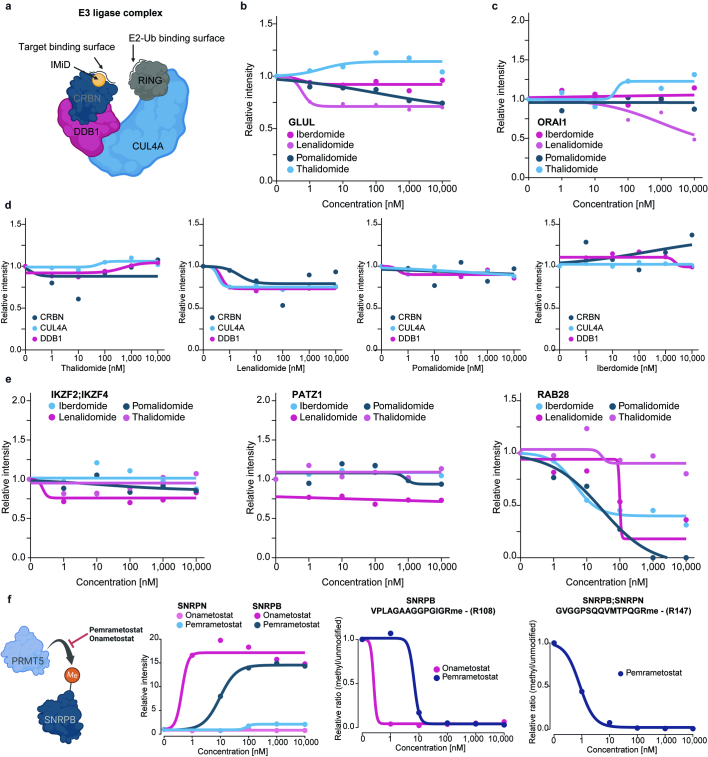
Molecular glues and PRMT5 inhibitors. **a**) Schematic representation of the RING-CUL4A-DDB1-CRBN complex with a bound IMiD molecular glue. **b, c**) Dose-dependent protein expression for GLUL and ORAI1 in response to several IMiDs. **d**) same as b) but for members of the RING-CUL4A-DDB1-CRBN complex. **e**) Same as b) but for IKZF2, PATZ1 and RAB28. **f**) Far left panel: schematic representation of SNRPB methylation by PRMT5 and its inhibition by pemrametostat and onametostat. Middle left panel: apparent upregulation of SNRPN and SNRPB by several PRMT5 inhibitors. Right two panels: Down-regulation of methylated Arg108 and Arg147 of SNRPB by PRMT5 inhibitors.

Even if not investigated here, we note that protein level changes induced by a drug may be cell-type specific. DecryptE profiling of immunomodulatory imide drugs (IMiDs) such as thalidomide, pomalidomide, lenalidomide and iberdomide did not show changes in protein levels for members of the E3 ligase complex itself (CRBN, DDB1, CUL4a) (Extended Data Fig. 8a,d). This suggests that the ubiquitinylation complex acts as a classic enzyme that releases its neo-substrates after ubiquitin transfer and that the molecular glue functions as a catalyst. Endogenous CRBN substrates (GLUL, ORAI1) were unaffected by IMiD treatment (Extended Data Fig. 8b,c). DecryptE profiles further showed that three of the four IMiDs degraded the neo-substrate IKZF1 in Jurkat cells in a dose-dependent fashion (Extended Data Fig. 4a). However, this was not the case for other reported neo-substrates including IKZF2, IKZF4 and PATZ1 (ref. ^38^). RAB28 was identified as a new neo-substrate of Iberdomide in Jurkat cells (Extended Data Fig. 8e)^39^. Such apparent discrepancies with the literature likely arise from molecular differences in the ubiquitinylation machinery present in a particular biological model system.

We note, that observed drug effects not only depend on the model system used but also on time, which might be different for each compound. This is supported by the vast differences in both the absolute number of regulations as well as which proteins show drug response when comparing decryptE profiles with published single dose data^22–24^ (Supplementary Fig. 2 and data deposited on MassIVE).

Future extensions of decryptE should include PTMs despite the fact that long-term drug responses may lead to complex PTM datasets that can be difficult to interpret. Special cases from the current work illustrating this need are pemrametostat and onametostat. Both are inhibitors of the protein arginine methyltransferase PRMT5 leading to reduced methylation levels of target proteins. By including methylation as a variable modification in a standard database search for protein identification, it was possible to measure in-cell inhibition of enzymatic activity with low nanomolar potency by monitoring methylation sites on the PRMT5 substrate SNRPB in response to the two drugs (Extended Data Fig. 8f). This would have gone unnoticed if the PTM level was not considered.

The perhaps most exciting pharmacological result of the present work is the observation that HDAC inhibitors led to strong and potent down-regulation of the TCR with a concomitant reduction of the ability to mount a T cell response. This may well explain the efficacy of HDAC inhibitors in the treatment of TCR signaling-driven T cell lymphoma or the attenuation of TCR signaling observed in animal models of certain autoimmune diseases^40,41^. At the same time, because TCR activity is critical for T cell lineage selection, antigen specificity, effector function and survival, a repressed expression of TCR complex components may turn out to be detrimental for the treatment of so-called ‘hot’ tumors that are characterized by immune cell infiltration, and which often respond to immune checkpoint inhibition therapy. In this context, clinical trial designs may be called into question that combine immune checkpoint inhibition with HDAC inhibitors^42^. However, there may also be beneficial scenarios. Persistent high antigen stimulation can lead to the phenomenon of T cell exhaustion, diminishing the ability of the immune system to fight a tumor^43–46^. In such cases, and depending on the immune status of the tumor, it may be possible that repressed expression of TCR complex components in response to HDAC inhibition reduces the absolute level of TCR stimulation to a degree that reinvigorates exhausted T cell responses. Clearly, further functional studies are required to better understand such possible HDAC inhibitor-related effects in patients and the potential of HDAC inhibitors as research tools in the context of studying T cell exhaustion.

Taken together, the results obtained in this study indicate that dose-dependent and proteome-wide measurements of drug-induced protein expression changes should become a standard tool alongside dose-dependent target deconvolution and pathway engagement studies. The combined information is highly valuable for basic research as well as preclinical and clinical drug discovery because it provides a better appreciation of the molecular capabilities of bioactive compounds, from chemical probes to human medicines.

## Methods

### Cell culture

Human Jurkat cells Clone E6.1 (ATCC TIB-152) were cultured in RPMI-1640 containing 10% fetal bovine serum (FBS) at 37 °C and 5% CO_2_. Culture medium was refreshed every 2–3 days and cells were kept at densities between 0.5 × 10^6^ and 2 × 10^6^ cells per ml until lysis or drug treatment.

Cell line authentication was accomplished by single nucleotide polymorphism profiling (Multiplexion).

### Compound information

The information of the target space of the 144 compounds included in this study was obtained from DrugBank Online (status of July 2023) and vendor specifications. Information about the clinical phase the compounds were in at the time the study was conducted was retrieved from ChemBL (status of July 2023).

### Compound treatment

Compounds were prediluted in DMSO and further in culture medium inside a 48-deep-well plate. Per 48-deep-well plate, three DMSO controls were added. For treatment, 4 × 10^6^ cells in RPMI-1640 medium supplemented with 10% FBS were added on top of each compound predilution resulting in a final volume of 2 ml and final treatment concentrations starting from 10 µM to 1 nM in full log_10_ steps, resulting in five doses for each drug (10 µM, 1 µM, 100 nM, 10 nM, 1 nM). Cells were incubated for 18 h if not stated otherwise at 240 rpm, 37 °C and 5% CO_2_. The following day, cells were subjected to viability assessment and lysis.

### Confluency, viability and metabolic activity assessment

For determination of cell viability and metabolic activity after compound treatment, 100 µl of cell suspension per well were added to a 96-well plate containing 50 µl of IncuCyte Cytotox Dye (250 nM final concentration, Sartorius) and alamarBlue Cell Viability Reagent (10% final concentration (v/v), Invitrogen). The plate was placed into the IncuCyte live-cell analysis system (37 °C and 5% CO_2_) and cells analyzed for cytotoxicity over a time course of 3 h (×10 magnification, scan type was standard with five images per well, channel selection was phase contrast and fluorescence (300 ms acquisition time), scan interval was every hour). The integrated software of IncuCyte (Basic Analyzer) was used for confluency and cytotoxicity analysis. After 3.5 h, metabolic activity was determined by fluorescence measurement of the AlamarBlue reagent using the fluorescence read out on the microplate reader FluoStar Omega (*λ*_ex_ = 544 nm and *λ*_em_ = 584 nm, BMG Labtech).

For confluency and metabolic activity evaluation, the resulting values were normalized to the average values for the DMSO control. For cytotoxicity, the values were corrected for differences in confluency, before normalizing the values to the average values for the DMSO controls. Dose–response curves were fitted to the data as described below (section ‘Curve fitting’).

Any microscopic pictures displayed in the paper or elsewhere were exported from the IncuCyte software as displayed and not further modified.

### Cell lysis for protein extraction

To obtain cell lysate from untreated cells (for optimization purposes), cell suspension was centrifuged at 172*g* for 5 min at room temperature, washed with PBS (phosphate buffered saline, without calcium or magnesium) and pelleted before resuspension in lysis buffer (2% SDS, 40 mM Tris/HCl, pH 8, 95 °C).

Lysis of compound-treated cells was performed in 96-deep-well plates. Therefore, after 18 h of treatment time, 48-deep-well plates were centrifuged (172*g*, 10 min, 4 °C), supernatant was discarded, cell pellets were resuspended in PBS and transferred to a 96-deep-well plate. Cell pellets were washed two more times with PBS and centrifuged to discard the supernatant before lysis in 100 µl of lysis buffer.

For hydrolysis of DNA, lysate was heated to 95 °C for 10 min while shaking at 172*g* and trifluoroacetic acid was added to a final concentration of 1% (v/v) and incubated for 1 min while shaking. Subsequently, *N*-methylmorpholin (NMM) was added for neutralization to the hot lysate to a final concentration of 2% (v/v). Lysate was stored at −20 °C until further use.

### Tissue and bacteria sample preparation

*Mus musculus* (*M. musculus*) and *Arabidopsis thaliana* (*A. thaliana*) tissue samples were snap frozen in liquid nitrogen before homogenization using the TissueLyser II (Quiagen, 5 min, 30 Hz, using one stainless steel bead with a 5 mm diameter). Lysis buffer (4% SDS, 40 mM Tris/HCl, pH 8) was added after removing the bead and samples were sonicated using the Bioruptor Pico (Diagenode, 25 cycles with 30 s on/off). DNA hydrolysis was performed as described above using final concentrations of 2% trifluoroacetic acid and 4% *N*-methylmorpholin, respectively. Lysates were cleared by centrifugation (60 min, 4 °C, 21,000*g*). Supernatant lysate was stored at −20 °C until further use.

*Escherichia coli* (*E. coli*) and *Pseudomonas aeruginosa* (*P. aeruginosa*) were grown in a shaker culture in Luria-Bertani medium at 37 °C, 300 rpm. When reaching an optical density of 0.5 and 0.6, respectively, cultures were harvested by centrifugation (172*g*, 60 min, 4 °C) and washed twice with PBS. Lysis buffer was added to the pellet, followed by DNA hydrolysis as described above. Lysate was sonicated using the Bioruptor Pico (above) before clearance by centrifugation (60 min, 4 °C, 21,000*g*). Cleared lysate was stored at −20 °C until further use.

### Isolation and sorting of T cells from healthy donors

Thrombocyte-depleted blood samples were obtained from two healthy, voluntary human donors (male, age 26) after they gave written and informed consent. This study was approved by a vote from the ethics committee of the University Hospital München rechts der Isar (564/18S). Sample were transferred into 50 ml Falcon tubes, with each tube containing approximately 15 ml of blood. The Falcon tubes were then filled up to a total volume of 37.5 ml with PBS, and the blood was thoroughly mixed. To isolate peripheral blood mononuclear cells (PBMCs), a 12 ml layer of Pancoll was meticulously underlaid using a 24 ml syringe with a long needle (G 20 × 2 3/4’; Ø 0.9 × 70 mm). Subsequently, the blood samples were subjected to centrifugation using a programmed gradient (acceleration of 7, deceleration of 1, 2, 7*g*, for 20 min at room temperature). Following the gradient centrifugation, the plasma fraction was discarded, and the PBMC-containing buffy coat was carefully collected. The PBMCs were then washed with 50 ml of PBS using centrifugation (441*g*, 5 min, at room temperature).

For cell separation, 10^7^ PBMCs were resuspended in 40 µl MACS buffer (PBS, 1% FCS, 2 mM EDTA) and incubated with 10 µl antihuman CD4 beads for 15 min at 4 °C. Subsequently, PBMCs cells were washed with 15 ml of MACS buffer and centrifuged. CD4 T cells were positively enriched with the autoMACS Pro Separator. Flowthrough was collected and used for the isolation of CD8 T cells according to the isolation protocol of CD4 T cells. Isolated primary T cells were cultured in RPMI-1640 containing 10% FBS and 1% penicillin and streptomycin (37 °C, 5% CO_2_) and were either subjected to HDACi treatment immediately or were activated as described below.

### HDACi treatment of peripheral T cells from healthy donors

For each population (CD4^+^/CD8^+^) a fraction of cells was activated using Dynabeads Human T-Activator CD3/CD28 for T Cell Expansion and Activation (Invitrogen) and incubated for 48 h (37 °C, 5% CO_2_) before HDAC inhibitor (HDACi) treatment. Naïve T cells were subjected to treatment immediately after isolation and sorting. Irrespective of activation status, cells were treated with different HDACi (five doses for each drug: 10 µM, 1 µM, 100 nM, 10 nM and 1 nM) for 18 h, followed by viability, confluency and cytotoxicity assessment as described above. Cell lysis, protein extraction followed by proteomic workflow and LC–FAIMS–MS/MS measurement was carried out as described in the respective sections. For samples, where available material was limited, protein input was adjusted for tryptic digestion and obtained peptides were loaded on Evotips and analyzed on an Evosep-FAIMS-Exploris set-up as described previously^46^ (for a full list of used instrument software, see Supplementary Table 3, Materials).

### Transcriptome sample preparation and analysis

For transcriptome analysis, Jurkat cells were treated according to the protocol described above. After 18 h, cells were lysed and total RNA was extracted using the ReliaPrep RNA Cell Miniprep System (Promega), according to the manufacturer’s protocol, and evaluated on a 2100 Bioanalyzer (Agilent Technologies). RNA library preparation occurred with the 3′ mRNA-Seq Library Prep Kit FWD with Unique Dual Indices (Lexogen) and was sent to Lexogen for gene expression profiling. Alignment of obtained reads was done using the data processing pipeline provided by the manufacturer using the QuantSeq FWD pipeline and *Homo sapiens* (*H. sapiens*) genome annotation. The obtained alignments were trimmed, reads were counted and normalized. Dose–response curves were fitted to the data as described below (section ‘Curve fitting’).

### SP3 sample preparation and tryptic digestion

Protein yield was determined by Thermo Pierce BCA (bicinchoninic acid) protein assays. All steps were performed according to the manufacturer’s protocol.

Before tryptic digest, detergent was removed by single-pot SP3 clean-up, following the protocol first described by Hughes et al.^25^ adapted to a Bravo Agilent liquid handling platform. In short, lysate containing 200 µg of protein was mixed with 1 mg SP3 beads (50:50 mixture of Sera-Mag carboxylate-modified magnetic bead types A and B (Cytiva Europe)) in a 96-deep-well plate and proteins were precipitated onto the beads in 70% ethanol in ddH_2_O (double distilled water).

The beads were washed three times with 80% ethanol in ddH_2_O and once with 100% acetonitrile (ACN). Disulfide bonds were reduced with 10 mM dithiothreitol for 45 min at 37 °C, followed by alkylation of cysteines with 55 mM CAA (2-chloroacetamide) for 30 min at room temperature in 100 µl of digestion buffer (2 mM CaCl_2_ in 40 mM Tris-HCl, pH 7.8). Trypsin (1:50 (wt/wt) enzyme-to-protein ratio) was added and proteins were digested off the beads at 37 °C and 1,200 rpm overnight. For peptide recovery, the beads were settled on magnets and the supernatant was transferred to a new 96-well plate. Beads were washed by addition of 100 µl 2% formic acid in ddH_2_O and the supernatant was transferred to the collection plate. Subsequently, the samples were desalted as described below.

### Desalting and drying of peptides

Before LC–MS/MS analysis samples were desalted using hydrophilic-lipophilic balanced (10 mg of *N*-vinylpyrrolidon-divinylbenzol porous particles 30 μm, Macherey-Nagel) 96-well plates using centrifugation at 7*g* for 1 min until specified otherwise. For this, hydrophilic-lipophilic balanced material was primed with 500 µl of isopropanol, ACN and solvent B (0.1% formic acid in 70% ACN in ddH_2_O) and equilibrated with 1,000 µl of solvent A (0.1% formic acid in ddH_2_O) before sample loading (by gravitation, 5 min). The sample flowthrough was reapplied to the plate and bound peptides were washed with 1,000 µl of solvent A. Peptides were eluted with 250 µl of solvent B (3 min, 7*g*; 1 min, 172*g*). Samples were frozen at −80 °C, dried by vacuum centrifugation and stored at −20 °C until LC–MS/MS measurement.

### High pH reversed-phase fractionation

Here, 50 µg of peptides (*A. thaliana* for Extended Data Fig. 1i and Jurkat for Fig. 3b and Extended Data Fig. 4d–e) were fractionated by basic pH reversed-phase material (reversed-phase sulfonate cartridge tips; 5 μl of polystyrene-divinylbenzene (PS-DVB) resin, Agilent) into six fractions using the Agilent AssayMAP Bravo pipetting system. The reversed-phase sulfonate cartridges were primed, washed and equilibrated according to the manufacturer’s protocol. Peptides were reconstituted in 100 μl of 25 mM ammonium formate (pH 10) and loaded onto the cartridges. Peptides were fractionated by increasing ACN concentrations (5, 10, 15, 20, 25, 30, 80%). The seven elution steps were either combined into six fractions, combining the 5 and 80% fractions, or into four fractions. For four fractions, the 5 and 25%, the 10 and 30%, the 15 and the 80%, and the 20% ACN fraction and the flowthrough were combined. All fractions were acidified with formic acid to a final concentration of 1%. Samples were frozen at −80 °C, dried by vacuum centrifugation and stored at −20 °C until LC–MS/MS measurement.

### Microflow-LC–(FAIMS)–MS/MS measurements

All samples (except where indicated otherwise) were analyzed on a microflow-LC–MS/MS system using a Vanquish Neo ultra high-performance LC system (Thermo Fisher Scientific) coupled to an Orbitrap Eclipse Tribrid mass spectrometer (Thermo Fisher Scientific) with or without installed FAIMS Pro Interface (Thermo Fisher Scientific). For a full list of used instrument software, see Supplementary Table 3, Materials.

Before measurement, samples were reconstituted in 0.1% formic acid, 2% ACN. For system optimization, the peptide concentration was determined using a Nanodrop system (Thermo Fisher Scientific) and the amount of peptide required for each run was injected accordingly. For drug profiling samples, half of the samples were injected per run (50 µg). For fractionated samples everything was injected.

Chromatographic separation was performed via direct injection on a 15 cm Acclaim PepMap 100 C18 column (2 µm, 1 mm inner diameter × 15 cm, Thermo Fisher Scientific) at a flow rate of 50 µl min^−1^. The column temperature was set to 55 °C. Solvent A was 0.1% formic acid in 3% DMSO in ddH_2_O, and solvent B was 0.1% formic acid and 3% DMSO in ACN. The gradients for different lengths can be found in Supplementary Table 3, LC gradients.

### Incorporation of FAIMS into microflow-LC–MS/MS

Because micro-LC separations generate much sharper peaks than nano-LC, the incorporation of FAIMS into microflow-LC–MS/MS system needed to be evaluated from the bottom up. We first characterized the device for peptide transmission at different compensation voltage (CV) values using a tryptic digest. With these data in hand, we next simulated how many and which CV values should be combined for best proteome coverage. Simulations were experimentally tested using LC gradient lengths between 15 and 180 min and we systematically compared performance with and without FAIMS. For gradient times of 15, 30 and 60 min, only one CV setting can be meaningfully used because CV switching takes substantial amounts of time. Regardless of LC times, FAIMS increased the number of identified protein groups at a given time or halved the MS time needed to obtain the same depth of analysis compared to the same LC set-up but without using FAIMS.

### Measurement without FAIMS installed

The OptaMax NG ion source (Thermo Fisher Scientific) with a heated electrospray ionization probe was used to acquire the data. The sprayer was positioned at middle position in the *x* axis (left to right), at position 1 in the *y* axis (front to back) and between positions M and L in the *z* axis (probe height).

The mass spectrometer was operated in data-dependent MS/MS, positive ion mode, using a spray voltage of 3.5 kV, a funnel radio-frequency lens value of 40, an ion transfer tube temperature of 325 °C and vaporizer temperature of 125 °C. The flow rates for sheath gas, auxiliary gas and sweep gas were set to 32, 5 and 0 l min^−1^, respectively.

A full-scan (MS1) was recorded from 360 to 1,300 *m/z* with a resolution of 120,000 in the Orbitrap in profile mode. The MS1 AGC target was custom set to 100% and the maxIT was set to 50 ms. Based on the full scans, precursors were targeted for the MS/MS scans (MS2) if the isotope envelope was peptidic (monoisotopic precursor selection), the charge was between 2 and 6 and the intensity exceeded 1 × 10^4^. The MS2 quadrupole isolation window was set to 0.4 *m/z*. Peptide fragmentation occurred in the ion routing multipole by HCD with a fixed collision energy mode, the collision energy normalized to the precursor *m/z* and charge with a collision energy of 28%. The MS2 scan was acquired in the Ion Trap with rapid scan rate in centroid mode and a defined first mass of 100 *m/z*. Specific MS2 properties as well as cycle times for different gradient length can be found in Supplementary Table 3, MS settings.

### Measurement with FAIMS installed

The same ion source and probe as above was used, applying the same position setting. The mass spectrometer was operated in data-dependent MS/MS, positive ion mode, using a spray voltage of 4 kV, a funnel radio-frequency lens value of 40, an ion transfer tube temperature of 325 °C and vaporizer temperature of 300 °C. The flow rates for sheath gas and auxiliary gas were set to 40 and 5 l min^−1^, respectively. FAIMS was operated with standard resolution (inner and outer electrode 100 °C) and a static carrier gas flow of 3.5 l min^−1^. Measurement parameters were unchanged and the respective FAIMS CV was set to the needed value. For measurements of drug perturbed samples, the 60 min gradient was used with a set CV of −30 V.

If more than one internal CV was used (system optimization), independent experiments were specified for the different CVs in the Tune method with the exact same settings, except for the different CV value (the used CV values can either be read directly from the figures or the raw file names). This leads to the MS looping through the specified experiments of the method, switching after each MS cycle (MS1 scan + MS2 scans). To keep the data points and thus quantification quality stable, the cycle time stated above was divided by the number of used internal CVs resulting in 0.75 s for 60 min (two CVs), 1.4 s for 120 and 180 min (two CVs) and 0.8 s for 120 and 180 min (three CVs).

### Database searching

The raw MS data files were processed with MaxQuant v.1.6.2.10 (ref. ^27^) using the integrated Andromeda search engine and searched against the respective reference database (*H. sapiens:* downloaded from UniProt containing canonical and isoforms 24 August 2020; 75,776 entries, *E. coli*: downloaded from UniProt containing canonical and isoforms 1 July 2021; 4,713 entries, *P. aeruginosa*: downloaded from UniProt containing canonical and isoforms 1 July 2021; 5,563 entries, *M. musculus*: downloaded from UniProt containing canonical and isoforms 1 July 2021; 25,381 entries, *A. thaliana*: Araport11 genome release downloaded from Arabidopsis.org containing canonical and isoforms 16 June 2020; 48,359 entries).

Raw files from runs with multiple internal FAIMS CVs had to be split into separate files based on CV values before MaxQuant searches. These separate files were specified as different fractions, as for the basic reverse-phase fractions, of the same experiment in MaxQuant. Multiple injections of the same sample were specified as the same experiment. Standard MaxQuant search parameters were used. Trypsin/P was specified as protease, allowing for up to a maximum of two missed cleavages. Carbamidomethylation of cysteine was specified as fixed modification, while oxidation of methionine and protein N-terminal acetylation were considered as variable modifications. Where specified, mono- and di-methylation of arginine and lysine was enabled as a variable modification. The label free quantification (LFQ) algorithm, with a standard LFQ minimum ratio count setting of 1, as well as the iBAQ (intensity-based absolute quantification) algorithm, with log fit, was switched on where needed. Where used, the Match-Between-Runs algorithm was switched on with default settings (0.7 min and 5 min for matching and retention time alignment window, respectively). The false discovery rate (FDR) was set to 1% on protein and peptide spectral match level. For Prosit rescoring, the FDR was set to 100% on protein and peptide spectral match level. The respective MaxQuant msms .txt and .raw files were rescored by Prosit. Peptides with *q* values ≤0.01 were retained and proteins were grouped based on the picked FDR method^47^. For MaxQuant output, proteins for which no unique peptide was found and thus where not distinguishable were aggregated to protein groups. For picked FDR protein group output, proteins are grouped on gene level and only unique peptides are considered. For readability, we refer to all only as proteins in the figures. Data analysis and visualization was performed using R (v.4.1.0) in RStudio (see Supplementary Table 3, Materials for full list of all packages used) and Microsoft Excel 365. Further editing of plots was done in Adobe Illustrator CS6. Information on whether a dataset was rescored or not can be found on MassIVE (Data availability section).

### Data processing and analysis

#### Curve fitting

For each protein–drug combination, the LFQ intensity relative to the average protein intensity in the DMSO controls was calculated for all drug concentrations. The same was done for each transcript–drug combination of the transcriptomic data using read counts. For the different viability metrics, the data were prepared as described above. To these normalized data, a sigmoidal four-parametric log-logistic model (equation (1)) was fitted using the dose–response curve R package (v.3.0-1), where *x* is the log_10_ of the drug concentration, *pEC*_50_ is the negative log of the inflection point of the curve (denoted as the effective concentration 50; EC_50_), *t* is the top or low-dose plateau, *b* is the bottom or high-dose plateau, *s* is the curve slope between the plateaus and *Y*(*x*) is the observed protein ratio compared to the vehicle control at concentration *x*.

1 $$Y\left(x\right)=\frac{t-b}{\left(1+{10}^{\left(s\times \left(x-{\mathrm{pEC}}_{50}\right)\right)}\right)}+b$$

For each resulting model, descriptive parameters were extracted and reported. Comprising the optimized slope (*s*), top (*t*), bottom (*b*) and inflection point (*EC*_50_), as well as the area under the curve, the coefficient of determination (*R*^2^), mean average deviation, the predicted *y* value of the fitted curve for the highest concentration (end of curve, fold change) and the slope of a linear model fitted to the data.

#### Curve classification

To avoid manual annotation of >1 million dose–response curves, a random forest classifier was trained using the ranger R package (v.0.14.1). As a ground truth dataset, curves of two compounds were manually annotated as up-, down- and nonregulated. The dataset was split into 80:20 for training and validation dataset, respectively (training 11,562, validation 2,883, total 14,409). The input features were comprised of the values described above, along with the relative LFQ intensities and number of unique peptides for all concentrations and abundance percentile of the respective protein in the DMSO control. After hyper parameter tuning, the final model was trained with 1,200 trees, randomly choosing 15 independent variables at each split and splitting only nodes with a minimum size of 3. Variable importance mode was set to impurity and the Gini split rule was applied. The model’s performance and quality were tested using the validation dataset, calculating precision, confusion matrices and ROC curves. The resulting classifier was used as a prefilter, plotting curves into separate PDFs and writing information into separate .txt files based on the predicted classes, thereby facilitating manual examination of all drug datasets. The same classifier was used for the dose–response curves of the drug perturbed transcriptome dataset. These regulated proteins were further analyzed to explore the mode of action of drugs.

#### Further filtering

For further analysis, a protein was regarded as up- or down-regulated if it was classified accordingly and the fold change exceeded 1.5 and 0.7 for up- and down-regulation, respectively. The same was applied to all transcripts, additionally retaining only observations where read counts were above 50 for all concentrations.

#### GO term enrichments

For the heatmap clustering of drugs with similar effects, a GO term enrichment analysis was performed for each drug individually using the clusterProfiler R package (v.4.2.2.)^48^. Each drug dataset was tested for enrichment of GO terms on all levels (cellular compartment, molecular function and biological process) both in up- and down-regulated proteins with the whole drug dataset as the background. *P* values were corrected using the FDR approach and the *q* value cut-off was set to 1. The enrichment results for up- and down-regulation were combined, retaining the more significant entry for duplications. After combining the enrichment results for all drugs, the *q* values were log transformed, multiplied by −1 for GO terms enriched in down-regulation and *z*-scored for each GO term individually. The heatmap depicts the combined, preprocessed GO term enrichment results after hierarchically clustering of both rows and columns using Pearson correlation as a distance metric and Weighted Pair Group Method with arithmetic mean as the agglomerative method. The GO term enrichment results displayed in Extended Data Fig. 6a were taken from the global GO term enrichment analysis described above. For Extended Data Fig. 5d a new GO term enrichment analysis was done (*P* value cut-off, 0.05; *P* value correction, FDR approach; Subontology, Molecular Function; whole *H. sapiens* database as background).

#### Dose-dependent methylation

The search results for lysine and arginine methylation were prepared for dose–response curve fitting similar to the process described for proteins and transcripts above. However, for each peptide–concentration–inhibitor combination the intensity ratio of methylated to unmethylated version was calculated. The resulting value in turn was then normalized to the respective DMSO control before continuing as described above (section ‘Curve fitting’).

#### Simulation of target coverage in relation to proteomic depth

For the simulation of target coverage over captured proteomic depth we ranked all >8000 proteins of this study by their mean iBAQ values in all DMSO controls in a descending fashion. To simulate the different proteomic depths, this list was cut at the indicated ranks (number of identified proteins). For each drug, we checked in turn how many of its targets were included in the resulting list and calculated the fraction of designated targets that were detected.

#### Replicate analysis

For the volcano plot displayed in Extended Data Fig. 2b assessing the quantitative reproducibility, the 48 DMSO controls were randomly assigned into two equally sized groups. After median centering normalization of the LFQ intensities of the picked FDR gene group output and filtering for completeness in the dataset, a two-sided Student’s *t*-test was performed for all 4,694 proteins. *P* values were corrected for multiple hypothesis testing using the FDR approach using the R package fdrtool (v.1.2.17).

For the comparison of quantitative reproducibility between unregulated and regulated proteins using the five individual doses for each inhibitor as replicates, the LFQ intensities of the picked FDR gene group output were normalized by median centering. The CoV was calculated across the five doses for each drug for each protein that was either classified as up- or down-regulated, or unregulated.

To assess the reproducibility of EC_50_ determinations, the curves for each protein for each drug replicate were fitted as described above. For proteins being classified as up or down-regulated in three out of four replicates per drug, the standard deviation of the pEC_50_s was calculated.

### Real-time RT–qPCR

For RT–qPCR analysis, cells were treated according to the protocol described above. After 18 h cells were lysed, and total RNA was isolated using the Monarch Total RNA Miniprep Kit (New England Biolabs) according to the manufacturer’s instructions. RNA yield was determined using the Qubit fluorometer (Thermo Fisher Scientific). Complementary DNA (cDNA) was generated from 2 µg of RNA from each sample using the LunaScript RT SuperMix Kit (New England Biolabs) according to the manufacturer’s protocol. Additionally, no-reverse transcriptase controls were generated for each sample during the reverse transcription step. After reverse transcription, the cDNA was diluted ~66 fold with nuclease-free ddH_2_O. qPCR was performed in triplicates on a CFX384 Touch Real-Time PCR Detection System (Bio-Rad Laboratories, Inc.) using 10 ng of cDNA per sample, the Luna Universal qPCR Master Mix (New England Biolabs) and the primer pairs as shown in Supplementary Table 3, Primer list. No-reverse transcriptase controls were measured in pools of all samples on each plate. Nuclease-free ddH_2_O was used as the nontemplate control for each assay. Cycling parameters were set to 95 °C (1 min), 40 cycles of 95 °C (15 s) and 60 °C (30 s with plate read on SYBR channel) each, and finally a melt curve was recorded from 60 to 95 °C with an increment of 0.5 °C per 5 s and SYBR channel plate reads after each increment. All samples treated with the same drug as well as the DMSO control were measured on the same plate.

### Analysis of RT–qPCR results

Quantification cycle (*C*_q_) and melting temperature (*T*_m_) values were determined in the CFX Manager v.3.1 software (Bio-Rad Laboratories, Inc.). The regression method of the software was used for *C*_q_ assessment with baseline correction and curve fit turned on. The fold change in expression after treatment and the ratio of truncated to full-length transcript were calculated in Microsoft Excel 365 from the mean *C*_q_ values for each sample using the 2^-∆∆Cq^ method^49^.

### T cell activation assay

Activation potential of HDACi treated Jurkat cells was analyzed using TCR and/or CD3 effector cells (nuclear factor of activated T cells or NFAT) from a T Cell Activation Bioassay (Promega) with slight adaptations of the manufacturer’s protocol. Briefly, TCR/CD3 effector cells (NFAT) were incubated with HDACi (five doses for each drug: 10 µM, 1 µM, 100 nM, 10 nM and 1 nM) for 16 h, followed by unspecific activation via CD3 and/or CD28 using the Human Anti-CD3/CD28 T Cell activation Kit (Cell Signaling Technology). After 5 h, the receptor-mediated signaling was read out by luciferase activity on a microplate reader FluoStar Omega (BMG Labtech). Thereby the strength of the luminescence signal corresponded to the strength of receptor-mediated signaling. To determine the strength of T cell activation, the luminescence signals were normalized to the DMSO control. Dose–response curves were fitted to the data as described in the section ‘Curve fitting’.

### T cell aggregation analysis

Using the bright light images of living activated human T cells, acquired using the IncuCyte live-cell analysis system as described above, cell aggregates were assigned and quantified (count and area in µm^2^). To this end images were processed by ilastik^50^, a supervised machine learning image analysis tool kit. The average aggregate size was calculated for each image by summing up the detected aggregate areas and dividing by the count of aggregates per image, treating the five images acquired per well as replicates. To assess statistical significance of the HDACi induced reduction of average aggregate size, an analysis of variance test was performed for each inhibitor individually, followed by a Tukey honest significant differences post hoc test.

### Reporting summary

Further information on research design is available in the Nature Portfolio Reporting Summary linked to this article.

## Online content

Any methods, additional references, Nature Portfolio reporting summaries, source data, extended data, supplementary information, acknowledgements, peer review information; details of author contributions and competing interests; and statements of data and code availability are available at 10.1038/s41587-024-02218-y.

## Supplementary information

Reporting Summary

Supplementary Tables Supplementary Tables 1–3.

Supplementary Figures Supplementary Figs. 1 and 2.

## Data Availability

The mass spectrometry proteomics raw data, UniProt reference databases (fasta files), MaxQuant search results, Prosit output, transcriptomics raw data and results, dose–response curve fitting outputs (.pdf and .txt files) and comparison to other studies have been deposited to the ProteomeXchange Consortium via the MassIVE partner repository with the dataset identifier MSV000093659 (PXD047799). All dose–response curves from this paper can be explored online in ProteomicsDB (www.proteomicsdb.org/decryptE). Additionally, dose–response curves can be visualized and compared in a custom-built Shiny App (https://decrypte.proteomics.ls.tum.de/). Additional information on cell morphology, cell metabolic activity, cytotoxicity, protein half-lives and protein targets of compounds and drug-target affinity (where available) are provided^10,51–53^ to help interpreting observed effects.

## Code availability

All code used for data curation, analysis and visualization is based on publicly available R packages as indicated in the respective section and will be made available upon request with no access restrictions.
