# Supplementary material for: Decrypting the molecular basis of cellular drug phenotypes by dose-resolved expression proteomics
Source: Nat Biotechnol. 2024 May 7;43(3):406–15. doi: 10.1038/s41587-024-02218-y (PMC11919725; doi:10.1038/s41587-024-02218-y)
Supplement: Supplementary file 1 — Reporting Summary [file 41587_2024_2218_MOESM1_ESM.pdf]

nature portfolio

Corresponding author(s): Bernhard Kuster

Last updated by author(s): Mar 8, 2024

Reporting Summary

Nature Portfolio wishes to improve the reproducibility of the work that we publish. This form provides structure for consistency and transparency in reporting. For further information on Nature Portfolio policies, see our Editorial Policies and the Editorial Policy Checklist.

Statistics

For all statistical analyses, confirm that the following items are present in the figure legend, table legend, main text, or Methods section.

n/a	Confirmed	
☐	☒	The exact sample size (*n*) for each experimental group/condition, given as a discrete number and unit of measurement
☐	☒	A statement on whether measurements were taken from distinct samples or whether the same sample was measured repeatedly
☐	☒	The statistical test(s) used AND whether they are one- or two-sided<br>*Only common tests should be described solely by name; describe more complex techniques in the Methods section.*
☒	☐	A description of all covariates tested
☐	☒	A description of any assumptions or corrections, such as tests of normality and adjustment for multiple comparisons
☐	☒	A full description of the statistical parameters including central tendency (e.g. means) or other basic estimates (e.g. regression coefficient) AND variation (e.g. standard deviation) or associated estimates of uncertainty (e.g. confidence intervals)
☐	☒	For null hypothesis testing, the test statistic (e.g. *F*, *t*, *r*) with confidence intervals, effect sizes, degrees of freedom and *P* value noted<br>*Give P values as exact values whenever suitable.*
☒	☐	For Bayesian analysis, information on the choice of priors and Markov chain Monte Carlo settings
☒	☐	For hierarchical and complex designs, identification of the appropriate level for tests and full reporting of outcomes
☐	☒	Estimates of effect sizes (e.g. Cohen's *d*, Pearson's *r*), indicating how they were calculated

Our web collection on statistics for biologists contains articles on many of the points above.

Software and code

Policy information about availability of computer code

Data collection	Vendor aquisition software was used provided with the Orbitrap Eclipse Tribrid mass spectrometer (Thermo Fisher Scientific): Tune (v.3.5), XCalibur (v.4.5) and the Orbitrap Exploris 480 mass spectrometer (Thermo Fisher Scientific): Tune (v.2.0), XCalibur (v.4.4). Sartorius IncuCyte S3 software (v 2009B) was used to aquire live cell imaging data. RT-qPCR Data was generated using the CFX Manager (v. 3.1)
Data analysis	MS raw data was searched using MaxQuant (v. 1.6.2.10). Data analysis was performed using R (v 4.1.0) in RStudio and Microsoft Excel 365. The following R packages were used: DRC (v. 3.0-1), ranger (v. 0.14.1), clusterProfiler (v. 4.2.2), data.table (v 1.14.2), stringr (v 1.5.0), tidyr (v 1.3.0), tidyverse (v 1.3.2), tidymodels (v 1.0.0), reshape2 (v 1.4.4), ggplot2 v 3.4.2, pathview (v 1.34.0), DOSE (v 3.20.1), ggpubr (v 0.6.0), wordcloud (v 2.6), enrichplot (v 1.14.2), fmsb (v 0.7.2), gridExtra (v 2.3), cowplot (v 1.1.1), ggrepel (v 0.9.3), grid (v 4.1.1), rqc (v 1.28.0), pheatmap (v 1.0.12), shinydashboard (v 0.7.2), hardhat (v 1.2.0), baguette (v 1.0.0), plotly (v 4.10.2), dplyr (v. 1.1.4), parallel (v. 4.1.1), ggplotify (v. 0.1.0), progress (v. 1.2.2), xlsx (v. 0.6.5), org.Hs.eg.db (v. 3.14.0), ggridges (v. 0.5.3), shiny (v. 1.6.0), shinyBS (v. 0.61.1), RColorBrewer (v. 1.1-2), DT (v. 0.2.1), readxl (v. 1.3.1), fdrtool (v. 1.2.17)

For manuscripts utilizing custom algorithms or software that are central to the research but not yet described in published literature, software must be made available to editors and reviewers. We strongly encourage code deposition in a community repository (e.g. GitHub). See the Nature Portfolio guidelines for submitting code & software for further information.

Data

Policy information about availability of data

All manuscripts must include a data availability statement. This statement should provide the following information, where applicable:

- Accession codes, unique identifiers, or web links for publicly available datasets
- A description of any restrictions on data availability
- For clinical datasets or third party data, please ensure that the statement adheres to our policy

The mass spectrometry proteomics raw data, MQ search results, Prosit output, transcriptomics raw data and results, and dose-response curve fitting outputs (.pdf and .txt files), as well as the reference databases used for database searching (downloaded from Uniport) have been deposited to the ProteomeXchange Consortium via the MassIVE partner repository with the data set identifier MSV000093659.
All dose-response curves from this paper can be explored online in ProteomicsDB (www.proteomicsdb.org/decryptE). Additionally, dose-response curves can be visualized and compared, in a custom-built Shiny App (https://decrypte.proteomics.ls.tum.de/). Addition information on cell morphology, cell metabolic activity, cytotoxicity, protein half-lives, and protein targets of compounds and drug-target affinity (where available) are provided to help interpreting observed effects.

Human research participants

Policy information about studies involving human research participants and Sex and Gender in Research.

Reporting on sex and gender	T-cells used in this study originated from 2 male donors. Sex- and gender-based analyses were not performed, because of too small sample number.
Population characteristics	Two male donors, both 26 years old, genotype na.
Recruitment	Voluntary, healthy donors were recruited for platelet donation and leftover blood was used for T-cells isolation after donors gave written and informed consent.
Ethics oversight	Ethics committee of the University Hospital München rechts der Isar (564/18 S)

Note that full information on the approval of the study protocol must also be provided in the manuscript.

Field-specific reporting

Please select the one below that is the best fit for your research. If you are not sure, read the appropriate sections before making your selection.

☒ Life sciences ☐ Behavioural & social sciences ☐ Ecological, evolutionary & environmental sciences

For a reference copy of the document with all sections, see nature.com/documents/nr-reporting-summary-flat.pdf

Life sciences study design

All studies must disclose on these points even when the disclosure is negative.

Sample size	No sample size calculations were performed. For the main proteomic screen 144 compounds were analyzed in 5 doses each with 6 DMSO control samples per 18 compound dose responses. These numbers were chosen to accomodate all 144 compounds on 8 96-well plates, enabling streamlined processing. Due to the superiority of dose resolved experiments these numbers are sufficient.
Data exclusions	Peptides with q-values >= 0.01 after PROSIT re-scoring were removed. Proteins that matched the contaminant database were treated likewise, see method section for details.
Replication	To determine reproducibility and robustness of the LC-MS/MS setup an endurance cycle consisting of 250 consecutive injections of 10 different samples was conducted prior to the proteomic screen. For the main screen, 48 DMSO controls were included along the entire time-frame of the 768 LC-MS/MS runs. For three compounds, dose-response profiling was done n = 4 times - see EDF1 for details. Dose-response compound profiling was done once for each drug for the main screen. All attempts of replication were successful.
Randomization	Compound distribution was randomized across all treatment plates. Different doses of the same compound were arranged sequentially on one plate in a column-wise fashion. Positions for DMSO controls were distributed across each plate and fixed across all plates. Processing of all samples on one plate was done in parallel.
Blinding	Investigators were not blinded to compound position on the treatment plate. This study would not be affected by blinding or bias.

Reporting for specific materials, systems and methods

We require information from authors about some types of materials, experimental systems and methods used in many studies. Here, indicate whether each material, system or method listed is relevant to your study. If you are not sure if a list item applies to your research, read the appropriate section before selecting a response.

Materials & experimental systems

n/a	Involved in the study
☐	☒ Antibodies
☐	☒ Eukaryotic cell lines
☒	☐ Palaeontology and archaeology
☒	☐ Animals and other organisms
☒	☐ Clinical data
☒	☐ Dual use research of concern

Methods

n/a	Involved in the study
☒	☐ ChIP-seq
☒	☐ Flow cytometry
☒	☐ MRI-based neuroimaging

Antibodies

Antibodies used

Antibodies for the T-cell activation assay were purchased from Cell Signaling Technology:
Human Anti-CD3/CD28 T Cell Activation Kit #70976 (CD3ε Activating Mouse mAb 1:500, CD28 Activating Mouse mAb 1:1000, Goat Anti-Mouse Kappa Light Chain, F(ab')2 Antibody 1:250)
Antibodies for activation of primary human T-cells were purchased from Invitrogen
Dynabeads™ Human T-Activator CD3/CD28 for T Cell Expansion and Activation (1 uL beads/1x10^5 cells)

Validation

All antibodies were validated by the vendor:
Human Anti-CD3/CD28 T Cell Activation Kit #70976: https://www.cellsignal.com/products/cellular-assay-kits/human-anti-cd3-cd28-t-cell-activation-kit/70976 Validated by CST by Western Blot using Jurkat cells
Dynabeads™ Human T-Activator CD3/CD28 for T Cell Expansion and Activation: https://www.thermofisher.com/order/catalog/product/11161D Validated by Thermo Fisher Scientific Baltics UAB using functional testing

Eukaryotic cell lines

Policy information about cell lines and Sex and Gender in Research

Cell line source(s)

Jurkat cells Clone E6.1 were derived from ATCC (TIB-152).

Authentication

Cell line authentication was accomplished by single nucleotide polymorphism (SNP) profiling (Multiplexion, Heidelberg, Germany).

Mycoplasma contamination

Cell lines were regularly tested negative for Mycoplasma contamination.

Commonly misidentified lines
(See ICLAC register)

No commonly misidentified lines were used.
