# Supplementary material for: Decrypting the molecular basis of cellular drug phenotypes by dose-resolved expression proteomics
Source: Nat Biotechnol. 2024 May 7;43(3):406–15. doi: 10.1038/s41587-024-02218-y (PMC11919725; doi:10.1038/s41587-024-02218-y)
Supplement: Supplementary file 3 — Supplementary Figs. 1 and 2. [file 41587_2024_2218_MOESM3_ESM.zip › SupplementaryFigure1.html]

Supplementary Figure 1


---

**Supplementary Figure 1:** Interactive HTML document showing the fraction of all
detected designated targets of drugs used in this study as a function of
the depth of the measured proteome (estimated using iBAQ ranks of the
DMSO controls). Each dot represents a drug. Horizontal lines, boxes and
whiskers of the boxplot depict the median, the range between the second
and the third quartile and 1.5-fold interquartile range fraction of
target proteins covered at the respective proteomic depth.
