# Supplementary material for: Decrypting the molecular basis of cellular drug phenotypes by dose-resolved expression proteomics
Source: Nat Biotechnol. 2024 May 7;43(3):406–15. doi: 10.1038/s41587-024-02218-y (PMC11919725; doi:10.1038/s41587-024-02218-y)
Supplement: Supplementary file 3 — Supplementary Figs. 1 and 2. [file 41587_2024_2218_MOESM3_ESM.zip › SupplementaryFigure2.html]

Supplementary Figure 2


## **a**

|  | This study | Mitchell et al. | Saei et al. | Ruprecht et al. |
| --- | --- | --- | --- | --- |
| Number of cell lines | 1 | 1 | 3 | 5 |
| Cell line(s) | Jurkat | HCT116 | A549, MCF-7, RKO | A549, Calu1, Calu6, 2030, 2122 |
| Number of drugs | 144 | 875 | 56, 9, 9 | 53 |
| Number of drugs overlapping with this study | NA | 33 | 12, 3, 3 | 13 |
| Treatment duration | 18 h | 24 h | 24 h | 24 h |
| Treatment concentration | Dose-resolved | 10 uM or 1 uM | LC50 after 48 h | LC50 after 72 h (A549, Calu6) or 96 h (Calu1, 2122, 2030) |

## **b**

---

**Supplementary Figure 2:** Interactive HTML document comparing the decryptE data set
to similar studies reported in literature. a) Tabular overview of the
key characteristics of the different data sets. b) Scatter plots
comparing the protein log2 fold changes of AZD-8055 treatment to DMSO
controls of the decryptE dataset (x-axis) to the respective comparative
study (y-axis). Each dot represents a protein. The different
concentrations of the decryptE data set are depicted side by side. Each
row is a comparison to another data set (cell line - study combination).
The key characteristics (cell lines, treatment duration and drug
concentration) are stated above each comparison. The dashed lines
represent a log2 fold change cut-off of 1. Different types of regulation
are highlighted by colors. For the different types of regulations and
regulation directions the number of proteins for this category are
displayed in the individual plots.
